# Generation of human long-lived plasma cells by developmentally regulated epigenetic imprinting

**DOI:** 10.26508/lsa.202101285

**Published:** 2021-12-24

**Authors:** Chester J Joyner, Ariel M Ley, Doan C Nguyen, Mohammad Ali, Alessia Corrado, Christopher Tipton, Christopher D Scharer, Tian Mi, Matthew C Woodruff, Jennifer Hom, Jeremy M Boss, Meixue Duan, Greg Gibson, Danielle Roberts, Joel Andrews, Sagar Lonial, Inaki Sanz, F Eun-Hyung Lee

**Affiliations:** 1 Division of Pulmonary, Allergy, Critical Care, and Sleep Medicine, Department of Medicine, Emory University, Atlanta, GA, USA; 2 Yerkes National Primate Research Center, Emory University, Atlanta, GA, USA; 3 Center for Vaccines and Immunology, Department of Infectious Diseases, College of Veterinary Medicine, University of Georgia, Athens, GA, USA; 4 Lowance Center for Human Immunology, Emory University, Atlanta, GA, USA; 5 Division of Rheumatology, Department of Medicine, Emory University, Atlanta, GA, USA; 6 Department of Microbiology and Immunology, School of Medicine, Emory University, Atlanta, GA, USA; 7 School of Biological Sciences, Georgia Institute of Technology, Atlanta, GA, USA; 8 Department of Hematology and Medical Oncology, Winship Cancer Institute, Emory University, Atlanta, GA, USA

## Abstract

Antibody secreting cells (ASCs) circulate after vaccination and infection and migrate to the BM where a subset known as long-lived plasma cells (LLPCs) persists and secrete antibodies for a lifetime. The mechanisms by which circulating ASCs become LLPCs are not well elucidated. Here, we show that human blood ASCs have distinct morphology, transcriptomes, and epigenetics compared with BM LLPCs. Compared with blood ASCs, BM LLPCs have decreased nucleus/cytoplasm ratio but increased endoplasmic reticulum and numbers of mitochondria. LLPCs up-regulate pro-survival genes *MCL1*, *BCL2*, and *BCL-XL* while simultaneously down-regulating pro-apoptotic genes *HRK1*, *CASP3*, and *CASP8*. Consistent with reduced gene expression, the pro-apoptotic gene loci are less accessible in LLPCs. Of the pro-survival genes, only *BCL2* is concordant in gene up-regulation and loci accessibility. Using a novel in vitro human BM mimetic, we show that blood ASCs undergo similar morphological and molecular changes that resemble ex vivo BM LLPCs. Overall, our study demonstrates that early-minted blood ASCs in the BM microniche must undergo morphological, transcriptional, and epigenetic changes to mature into apoptotic-resistant LLPCs.

## Abstract (precis)

This study shows that the generation of human long-lived plasma cells requires blood antibody secreting cells to undergo epigenetic and transcriptional reprogramming in response to the bone marrow microniche to become apoptosis resistant and survive to secrete antibodies for a lifetime.

## Introduction

Human long-lived plasma cells (LLPCs) provide neutralizing antibodies during infection and can safeguard against subsequent encounters for a lifetime (Amanna et al, 2007; Halliley et al, 2015). These cells are quiescent, terminally differentiated, non-dividing, and persist after infection or vaccination in the BM in humans, mice, and nonhuman primates (Slifka et al, 1998; Halliley et al, 2015; Hammarlund et al, 2017). Although the heterogeneity of human BM antibody secreting cell (ASC) subsets has been described (Medina et al, 2002; Gonzalez-Garcia et al, 2006), the human LLPC compartment was identified in the BM as CD19^−^CD38^hi^CD138^+^ from adults who retained ASCs with virus-specificities (i.e., measles and mumps) within this subset after childhood viral infections decades earlier (Halliley et al, 2015). Furthermore, Hammarlund et al (2017) recently demonstrated the persistence of LLPC in the BM for 10 yr after tetanus vaccination, thereby providing direct evidence of the longevity of LLPCs in the BM. Whether ASCs were terminally differentiated as LLPCs in the blood immediately after differentiation or required further maturation in the BM was not clear.

Interestingly, there was evidence that early-minted blood ASCs, or plasmablasts, appear in the circulation 5–8 d then quickly disappeared after vaccination (Halliley et al, 2010) only to be found in the LLPC compartment years later (Halliley et al, 2015). However, interrogation of the BM within 21 d after immunization found vaccine-specific ASCs in both the LLPCs (pop D) and the BM subset CD19+CD38hiCD138+ (pop B) suggesting ongoing BM maturation (Halliley et al, 2015). In all, nascent blood ASCs migrate to the BM in multiple compartments early after vaccination to be located only in the LLPC compartment years later.

Whether the selected circulating ASCs merely migrate to privileged BM microniches to establish residence and/or undergo further molecular changes upon arrival in the microniche had not been studied. A major challenge for addressing this fundamental question has been that mouse and human ASCs undergo rapid apoptosis ex vivo (Wols et al, 2002; Cassese et al, 2003; Nguyen et al, 2018b). To overcome this limitation, we developed a novel in vitro ASC survival system that mimics the human BM michroniche which contains a combination of soluble factors secreted from primary BM mesenchymal stromal cells, exogenous APRIL, and hypoxic conditions (Nguyen et al, 2018a). In essence, this unique in vitro ASC culture system mimics the human BM’s ability to sustain ASCs in culture for 56 d and provides an essential tool to study further maturation of these cells.

Here, we compared the morphology, transcriptomes, and chromatin accessibility of blood ASCs and BM LLPCs ex vivo*.* We show that these subsets have distinct morphology and subcellular features with changes in nuclear/cytoplasm ratios, increased ER, and greater numbers of mitochondria. Multiple pathways undergo transcriptional and epigenetic changes, and BM LLPCs become refractory to apoptosis by decreasing expression and accessibility of pro-apoptotic genes to maintain survival. To demonstrate that the external cues from the BM microniche played a role in this transformation, we cultured early minted ASCs in the in vitro BM mimetic and observed similar morphologic, transcriptomic, and epigenetic changes, thereby validating blood ASC maturation into an LLPC. Altogether, this study illustrates that upon arrival to the BM microniche, early-minted blood ASCs undergo transcriptional and epigenetic modifications to become resistant to apoptosis and mature into an LLPC.

## Results

### Human blood and BM ASCs are morphologically different

**Figure 1.**
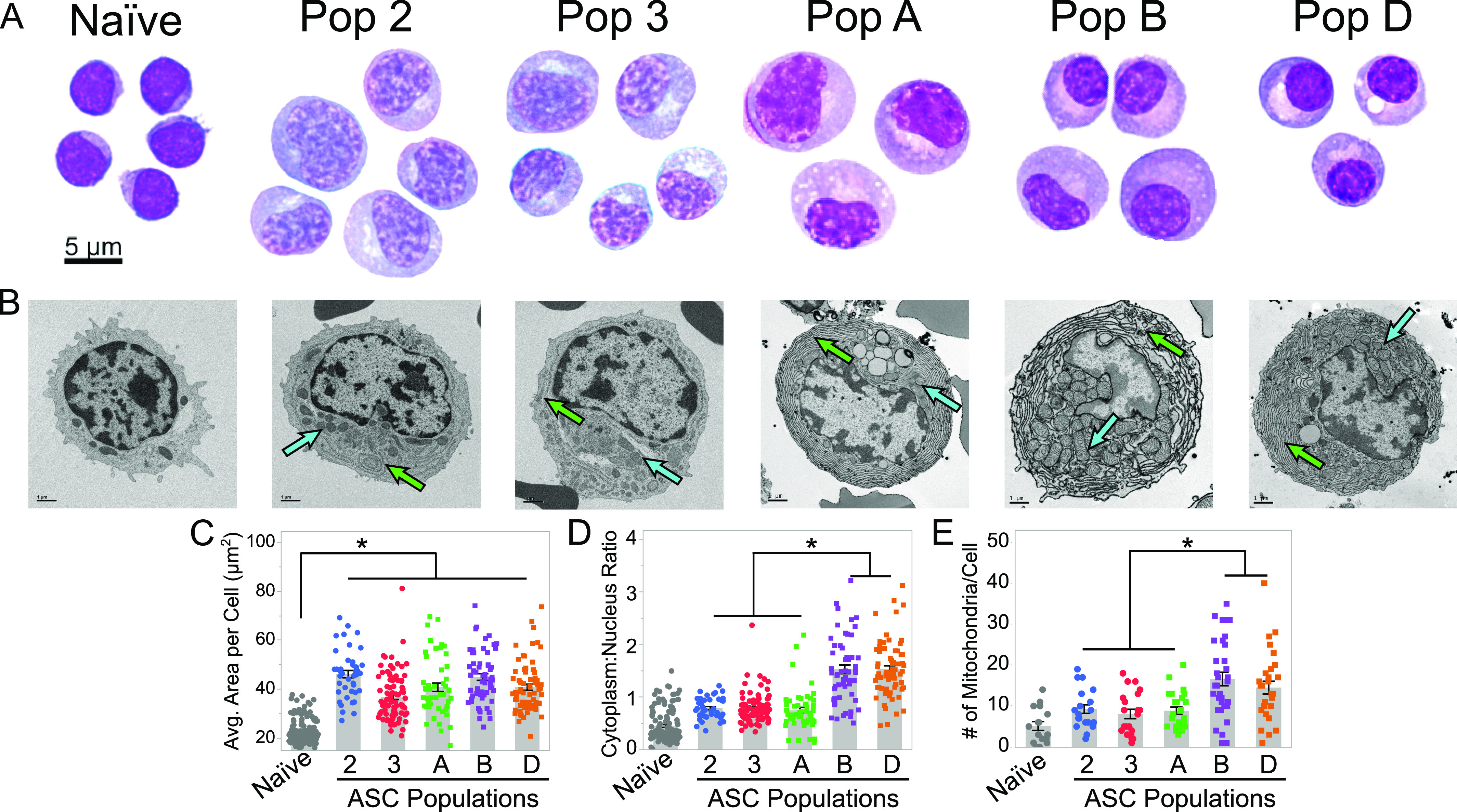
Human blood and BM antibody secreting cells (ASCs) have different morphology and subcellular structures. **(A)** Representative Wrights-Giemsa–stained human naïve B cells, blood ASC populations 2 and 3, and BM ASC populations A, B, and D from at least three independent donors. Scale bar is applicable to all images. **(B)** Representative transmission electron microscopy images of naïve B cells and ASC populations from two to three independent samples per population. Green and blue arrows indicate ER and mitochondria, respectively. Scale Bar = 1 μm. **(C, D)** Comparison of the average area per cell (C) and cytoplasm to nucleus area ratio (D) for each ASC population and naïve B cells from at least six independent blood and four independent BM samples. Statistical significance was assessed using a linear mixed-effect model with Tukey–Kramer HSD post hoc analysis. **(E)** Quantification of the number of mitochondria per cell transmission electron microscopy images. Statistical significance was assessed using a generalized linear model using a background Poisson distribution with chi-square post hoc analysis. Asterisks indicate statistical significance, *P ≤ 0.05 in all panels. Gray bars = mean; error bars = SEM.

**Figure S1.**
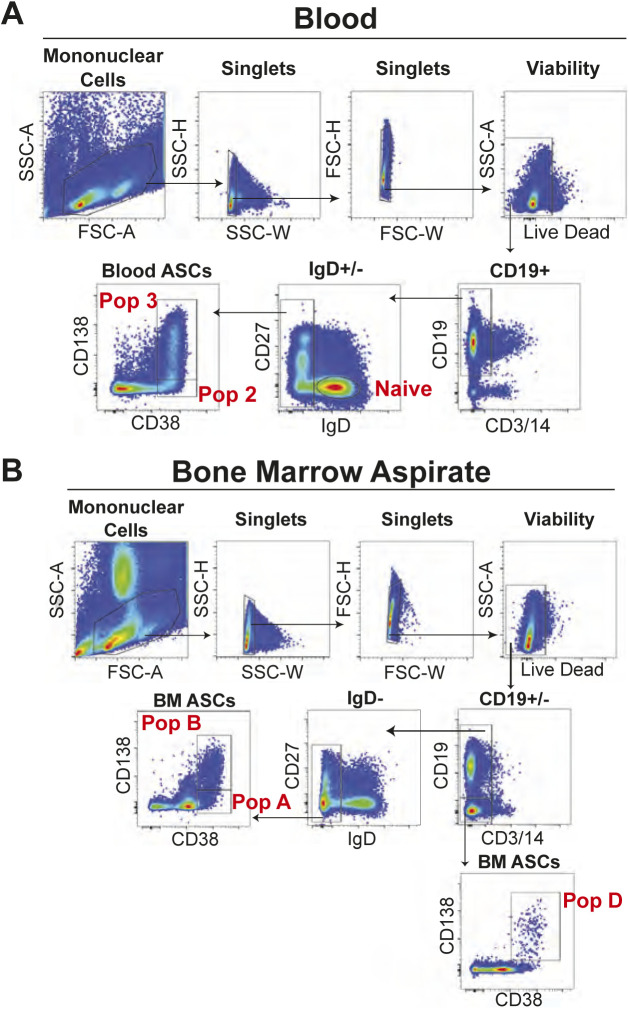
Blood and BM antibody secreting cell (ASC) FACS gating strategies for ex vivo analyses. **(A)** Fluorescence activating cell sorting strategy for enriching blood ASC Pop 2 (CD19+CD38++CD138−) and Pop 3 (CD19+CD38++CD138+). **(B)** Fluorescence activating cell sorting strategy for enriching BM ASC populations Pop A (CD19+CD38++CD138−), Pop B (CD19+CD38++CD138+), and Pop D (CD19−CD38++CD138+).

Previous studies compared human blood and BM ASC subsets within each compartment (Halliley et al, 2015; Garimalla et al, 2019) but did not address whether blood and BM ASCs were morphologically different. Here, we compared the morphology and ultrastructure of human blood ASC populations two (CD19+CD38++CD138−) and three (CD19+CD38++CD138+) from healthy donors after vaccination with BM populations A (CD19+CD38++CD138−), B (CD19+CD38++CD138+), and D (CD19−CD38+CD138+), the human LLPC from healthy donors (Figs 1A and B and S1A and B). Naïve B cells (CD19+IgD+CD27−) were also isolated from blood as controls. As expected, all ASC subsets were larger than naïve B cells (Fig 1C). There was considerable heterogeneity in the average area of ASCs within each population, and thus, no difference in size were identified (Fig 1C). The cytoplasm to nucleus ratio was significantly higher in Pop B and D (Fig 1D), and interestingly, the nuclei of Pop B and D (the CD138+ ASC in the BM), appeared rounder and more condensed compared with Pop A and blood ASC subsets (Fig 1A).

We performed transmission electron microscopy (TEM) to determine differences in subcellular structures. Compared with naïve B cells, all ASCs appeared larger and possessed more mitochondria and ER, consistent with the primary ASC function to synthesize and secrete antibody (Fig 1B). To our surprise, the ER content in blood ASCs were less than in CD138+ and CD138− BM ASCs, suggesting that blood ASCs increase ER mass when becoming a BM ASCs (Fig 1B). In addition, CD138+ BM ASC had higher numbers of mitochondria per cell compared with CD138− BM ASCs or blood ASC subsets, suggesting potential differences in metabolism between blood and BM ASCs (Fig 1E).

### Human blood and BM ASCs are transcriptionally distinct

**Figure 2.**
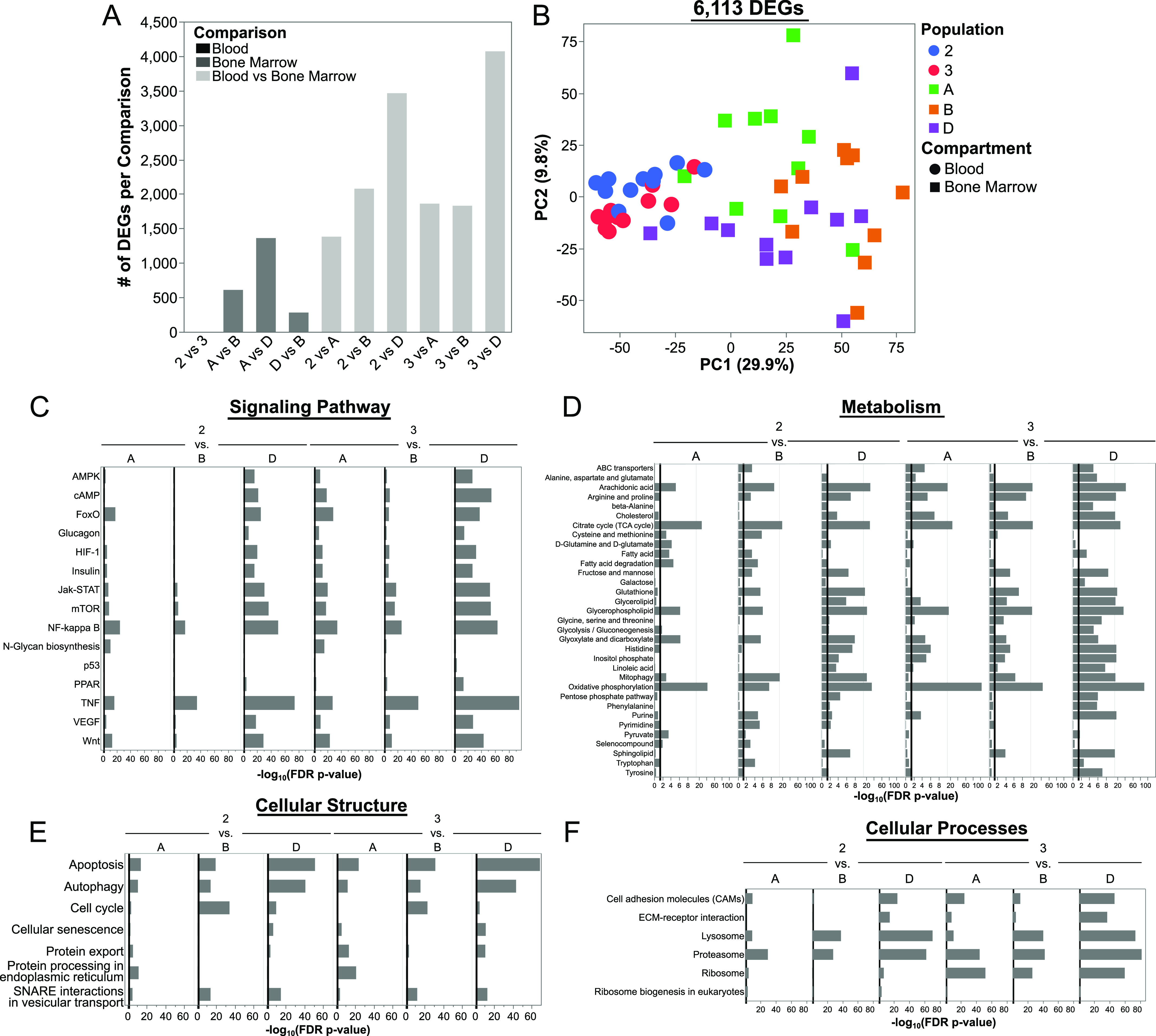
Human blood and BM antibody secreting cells (ASCs) are transcriptionally distinct. **(A)** Bar graph indicating the number of differentially expressed genes (DEGs) per comparison. **(B)** Principal components analysis using DEGs identified between human blood and BM ASC populations. DEGs were identified using a linear-mixed effect model with Tukey–Kramer post hoc analysis followed by Benjamini–Hochberg false discovery rate correction of each *P*-value. Genes with a false discovery rate–corrected *P*-value ≤ 0.05 between at least one comparison are considered differentially expressed and are used in the analysis. **(C, D, E, F)** Pathway enrichment analysis using all the gene expression data irrespective of differential expression. Selected KEGG pathways that are different between at least one pairwise comparison of ASC populations are shown. Pathways were classified into functional categories for data presentation.

We profiled the transcriptome of blood (Pop 2 and 3) and BM (Pop A, B, and D) ASC subsets represented by 57 samples from 11 healthy adults after tetanus vaccination and 11 BM from steady state (Figs 2 and S1 and Table S1). The blood and BM samples were not from the same individuals.

Table S1 Patient information for all analyses.

As expected, the blood ASC Pop 2 and 3 were transcriptionally similar despite their differential surface expression of CD138 (Garimalla et al, 2019) (Fig 2A). Similarly, Pop A and Pop D had the largest number of differentially express genes (DEGs) between BM populations (Halliley et al, 2015) (Fig 2A). Notably, the CD138+ BM subsets Pop B and D, were more similar transcriptionally to one another than to the CD138− Pop A (Fig 2A), as previously shown (Halliley et al, 2015). There were 6,113 DEGs among any pair-wise comparison with the greatest number of DEG occurring between compartment comparisons. For example, blood and BM ASC subsets had more transcriptional differences that ranged from 1,386 to 4,075 DEGs than any pair-wised comparison of blood or BM ASC subsets (Fig 2A). The blood ASC subsets were closest transcriptionally to Pop A and B in the BM, and the least similar to LLPCs in Pop D (Fig 2A). In agreement with the number of DEGs between the populations, the major variance of the principal components analysis (PCA) was the ASC was isolated from blood or BM (PC1 29.9%, Fig 2B). PC2 accounted for 9.8% of the variation in gene expression and mostly resolved Pop A from Pops B and D in the BM (Fig 2B).

There were numerous pathways that were different between blood and BM ASCs (Fig 2C–F). Despite Pop 2 and 3 being nearly identical by DEGs, the pathway analysis showed that Pop 3 versus BM ASCs contained more differences than Pop 2 versus BM ASC comparisons (Fig 2C–F). Nevertheless, the largest pathway differences were found between blood ASCs versus Pop D (LLPCs). A representative list of differentially enriched pathways included those in signaling pathways (mTOR, HIF1, VEGF, TNF, and NF-κ B, and Jak-STAT; Fig 2C) and metabolism pathways (mitophagy, ABC transporters, oxidative phosphorylation; Fig 2D). Pathways involved with changes in cellular structures between ASC subsets included the proteasome, lysosome, and cell adhesion molecules (Fig 2E). Last, cellular processes that are important for LLPC maturation included protein export, autophagy, and apoptosis (Fig 2F). Together, these results show that human blood ASC subsets have significantly different transcriptional profiles compared with BM ASCs indicating blood ASCs undergo transcriptional changes to become a LLPC.

### Human blood and BM ASCs are epigenetically distinct

**Figure 3.**
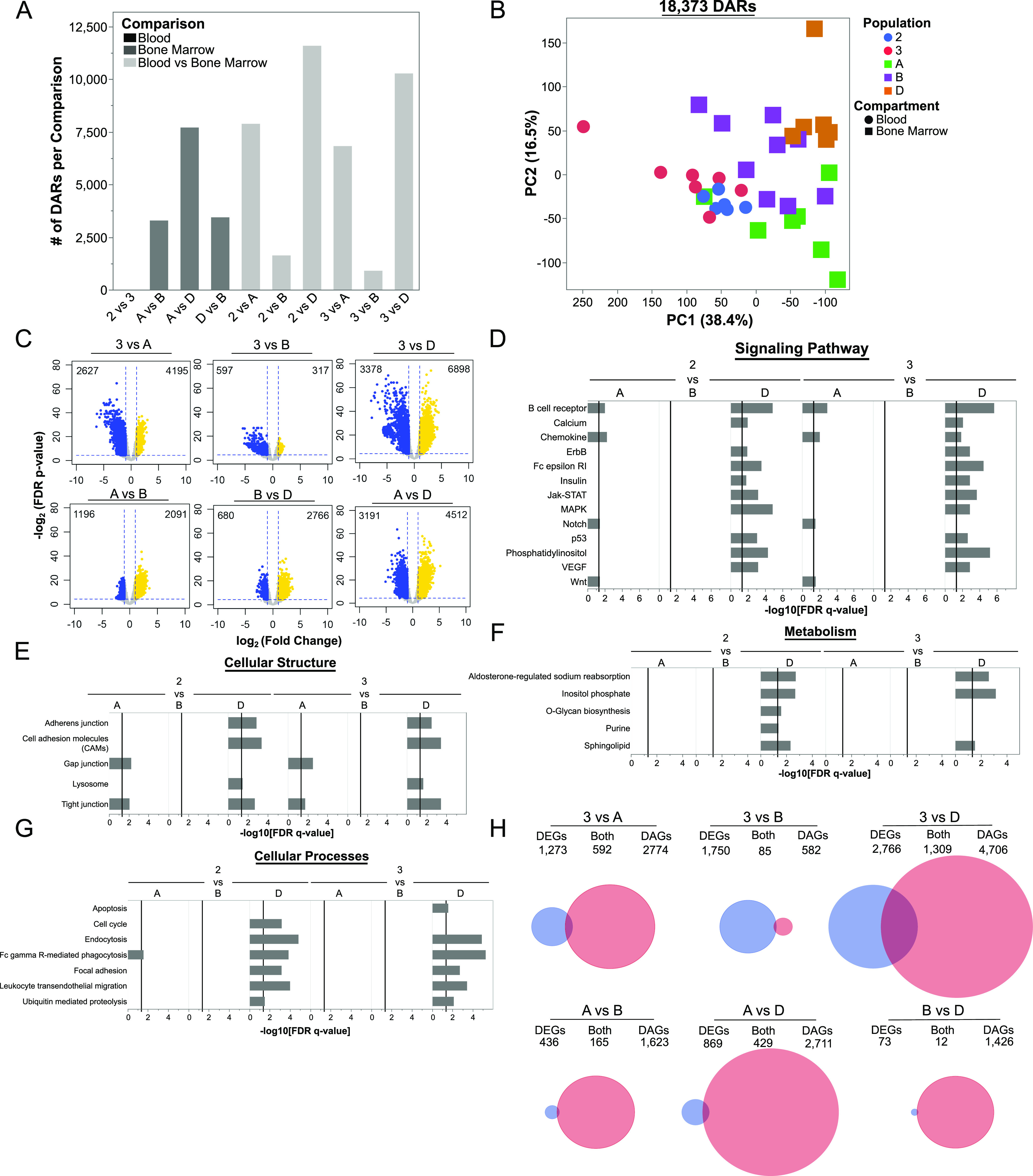
Epigenetic states of human blood and BM antibody secreting cell populations are different and suggest maturation relationships. **(A)** Bar graph showing the number of differentially accessible chromatin regions (DARs) in each pairwise comparison. Peaks were compared between populations and deemed a DAR if the peak displayed an absolute log_2_ fold change (log_2_FC) > 1 and a Benjamini–Hochberg false discovery rate–corrected *P*-value < 0.05 between the populations. **(B)** Principal components analysis using DARs identified between human blood and BM antibody secreting cell populations. **(C)** Volcano plots indicating the number of up-regulated (yellow) and down-regulated (blue) per comparison. Additional plots with Pop 2 comparisons are in Fig S2. **(D, E, F, G)** Gene set enrichment analysis using DARS form pairwise comparisons. Pathways were classified into categories for data presentation purposes. **(H)** Venn diagrams showing the overlap of differentially expressed and accessible genes in blood and BM comparisons (top) and in BM and BM comparisons (bottom). Circles are scaled to proportion of differentially expressed genes (DEGs) and DAGs in all shown comparisons. Additional diagrams comparing to Pop 2 can be found in Fig S3.

**Figure S2.**
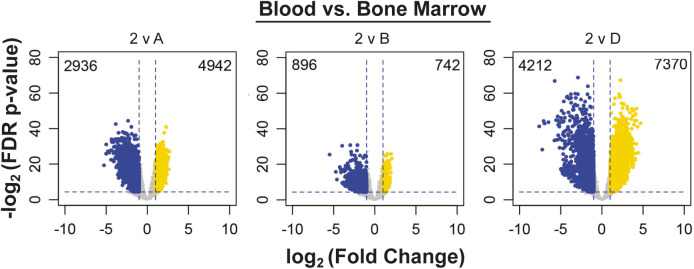
Volcano plots of DARs in Pop 2 and BM ASC population comparisons. Volcano plots show the number of differentially up-regulated (yellow) and down-regulated (blue) per indicated comparison.

To investigate if there were distinct chromatin signatures between the blood and BM ASC subsets, we performed matching ATAC-sequencing of 38 samples from the blood and BM ASC samples described above (Table S1). There were 18,373 differentially accessible chromatin regions (DARs) between blood and BM subsets that mapped to 8,944 genes (Fig 3A and B). Similar to the RNA-Seq, PC1 accounted for 38.4% of the variance in the DARs and predominantly separated the blood and BM ASC populations; PC2 resolved the BM ASC populations from one another (Fig 3B). Similar to their transcriptional profiles, Pops 2 and 3 had nearly identical chromatin accessibility with three DARs in *LAPTMA4*, *SDC1* (*CD138*), and *UGT8* loci. Comparisons between the BM ASC subsets followed trends similar to the transcriptome profiles with the largest number of DARs between Pop A and D (7,717 DARs), Pop A and B with 3,302 DARs, and the least between Pop B versus D (3,452 DARs) (Fig 3A). Interestingly, Pops 2 and 3 shared few DARs with Pop B (1,644 and 916 DARs, respectively) indicating that chromatin accessibility of blood ASCs is most similar to Pop B versus Pop A or LLPCs (Figs 2A and 3B). Whereas significant chromatin accessibility changes were occurring between blood and BM populations, more DARs were closed in Pop D compared with blood and other BM ASC subsets (Figs 3C and S2). On the other hand, Pop A had DARs that were more accessible compared to both pop B and D (Fig 3C). In all, these differences indicate that blood ASCs undergo further epigenetic changes to become LLPCs after migrating to the BM.

We performed gene set enrichment analysis using genes that contained a DAR (i.e., differentially accessible genes or DAGs) to understand pathways represented by the genes undergoing accessibility changes. There were no statistically different pathway enrichments between Pop B versus blood ASCs because of the limited number of DARs (Fig 3A and D–G). Some ATAC-seq pathways overlapped with transcriptional analysis such as apoptosis, Jak-STAT signaling, and lysosome pathways, highlighting the importance of the coordinated epigenetic and transcriptional regulation of these pathways (Fig 3D). Additional pathways that overlapped included signaling pathways (e.g., VEGF, phosphatidylinositol, and p53; Fig 3D), cellular structure (e.g., cell adhesion molecules; Fig 3E), cellular metabolism (e.g., inositol phosphate; Fig 3F), and cellular processes (e.g., focal adhesion, phagocytosis, endocytosis, and apoptosis; Fig 3G).

**Figure S3.**
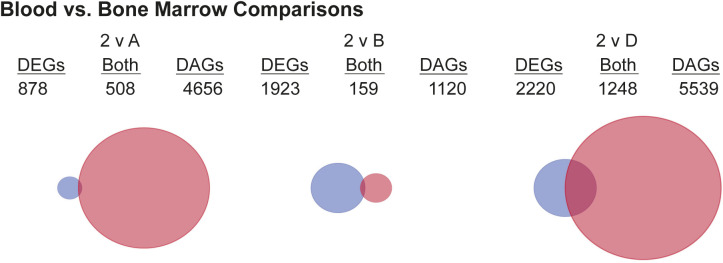
Venn diagrams of differentially accessible (DAG) and expressed (DEG) genes between Pop 2 and BM antibody secreting cell populations. Venn diagrams showing overlapping DEGand DAG in the indicated comparisons. Circles are scaled to proportion of DEGs and DARs.

Given the pathway overlap in transcriptional and epigenetic analyses, we compared the DAGs with the DEGs (Fig 3E). Because pop 2 and 3 were similar, we only show comparisons with Pop 3 (Pop 2 comparisons in Fig S3). As expected, genes were the most transcriptionally and epigenetically concordant (i.e., overlap) between Pop 3 versus D (Fig 3H). However, the transcriptional (DEG in blue) and epigenetic (DAG in red) changes were mostly discordant. Among the BM ASC subsets, chromatin accessibility changes (red) were most prominent, suggesting that later stages of LLPC maturation involve chromatin remodeling.

### LLPCs become resistant to apoptosis through epigenetic and transcriptional regulation of pro-survival and pro-apoptotic proteins

**Figure 4.**
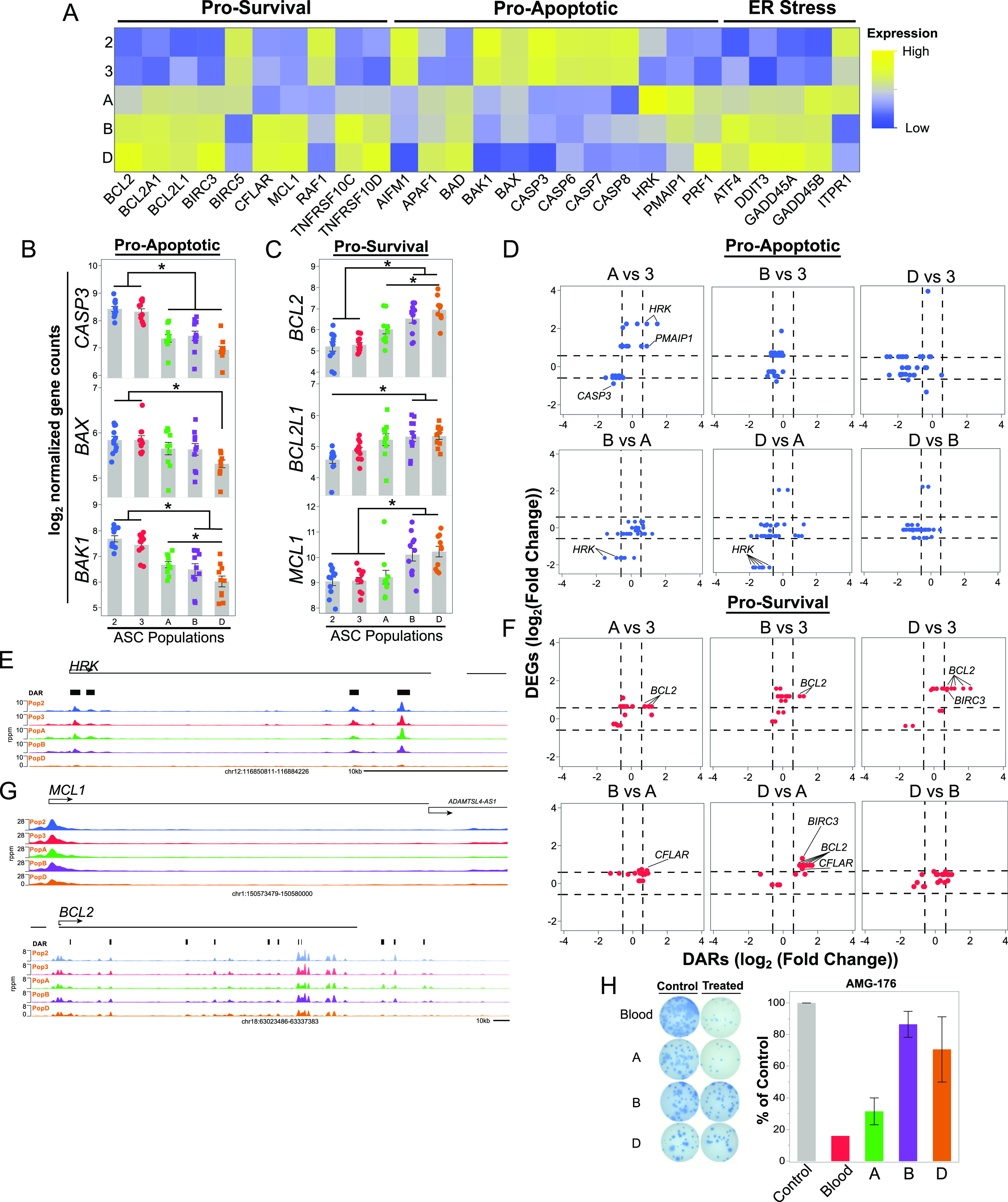
The apoptosis pathway is transcriptionally and epigenetically regulated in human and blood and BM antibody secreting cell (ASC) populations. **(A)** Gene expression patterns of selected genes annotated in the KEGG apoptosis pathway in blood and BM ASC populations. Apoptosis pathway genes were classified as ER-stress, Pro-Survival, and Pro-Apoptotic for presentation. **(B, C)** Gene expression of pro-apoptotic genes *CASP3*, *BAX*, and *BAK1* (B) and pro-survival genes *BCL2*, *BCL2L1*, *and MCL1* (C) in blood and BM ASC populations. Significance determined as in Fig 2. **(D, F)** Coordinated changes in gene expression and accessibility changes of pro-apoptotic (D) and pro-survival (F) genes identified in Fig 4A. Additional comparisons are available in Fig 4. Genes are considered concordant if they have a log_2_ fold change in accessibility and gene expression of less than or greater than 0.585 (dashed lines), which is equivalent to a fold change of 1.5. **(E, G)** ATAC-seq accessibility profile for *HRK*, *MCL1*, and *BCL2*. rppm = reads per peak per million. **(H)** Total IgG secretion of blood and BM ASC populations measured by ELISPOT with or without exposure to 0.2 μM AMG-176, an MCL1 inhibitor. Percent IgG graphed normalized to the number of spots in the control, which was untreated or treated with 0.1% DMSO. For all panels, asterisks indicate statistical significance, *P ≤ 0.05.

We found that the apoptosis pathway had concordant transcriptional and epigenetic changes between blood and BM ASCs making apparent the intuitive importance of survival in LLPC. We found higher expression in pro-apoptotic genes (e.g., *CASP3*, *CASP8*, *BAK1*, *BAX*, and *AIFM1*) in the blood ASC subsets compared with the BM subsets, but interestingly, some pro-apoptotic genes such as *PMAIP1* and *HRK*, were also elevated in Pop A (Fig 4A and B). In contrast, pro-survival gene expression (e.g., *MCL1*, *BCL2*, *BCL2L1*, and *BIRC3*) was typically highest in the CD138+ BM Pops B and D (Fig 4A and C). As a protein secretory factory, ASCs upregulate the unfolded protein response as a cellular stress response related to ER stress to handle the unfolded or misfolded proteins in the ER lumen. Genes involved in ER stress (e.g., *GADD45A*, *ATF4*, and *DDIT3*) were up-regulated in BM ASCs with the highest expression in Pops B and D (Fig 4A). Moreover, *ITPR1* had lower expression in BM ASCs compared with blood ASCs. *ITPR1* are ion channels that regulate the release of calcium sequestered in the ER into the cytoplasm, thereby triggering the intrinsic apoptosis pathway (Fig 4A). Hence, BM ASCs, particularly LLPCs, minimize pro-apoptotic while up-regulating pro-survival gene expression.

**Figure S4.**
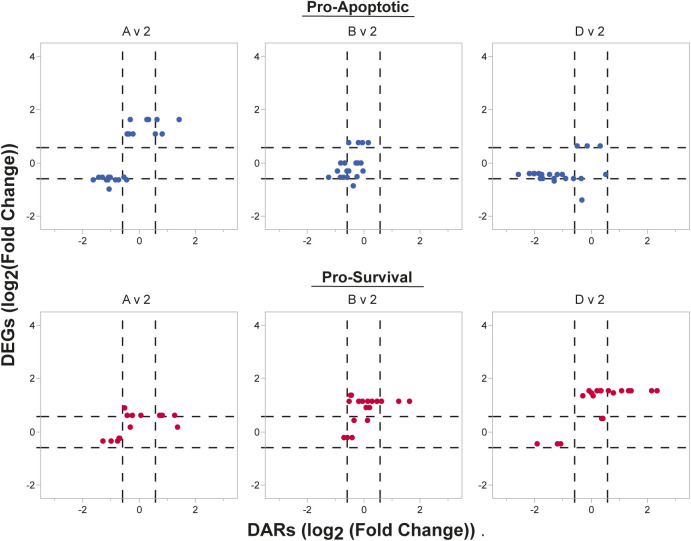
Additional concordance analysis comparisons of apoptosis-related genes between Pop 2 and BM antibody secreting cell populations. Coordinated changes in gene expression and accessibility of pro-apoptotic and pro-survival genes. Genes are considered concordant if they have a log_2_ fold change in accessibility and gene expression of less than or greater than 0.585 (dashed lines), which is equivalent to a fold change of 1.5.

Although LLPCs alter their gene expression to be refractory to apoptosis, it was unclear if this phenotype was due to mere transcriptional changes, epigenetic alterations or both. Although the pathway analysis noted overlapping differences by RNA-seq and ATAC-seq in the apoptosis pathway, there were few significant concordant transcriptional and epigenetic changes in apoptosis-related genes (Fig 4D and F). Even though many of the pro-apoptotic genes were not labeled as concordant because of the arbitrary cutoff, these genes were often associated with closed gene loci (Figs 4D and S4). HRK and CASP3 expression and accessibility changed concordantly (up and open) in the Pop A versus BM populations and (down and closed) in Pop D versus blood populations (Fig 4D). HRK expression increases during kinase inhibition and/or growth factor withdrawal and sensitizes a cell to initiate apoptotic programming by inhibiting the pro-survival functions of BCL-xL/BCL2L1 (Singh et al, 2019). Thus, we show that, HRK is concordantly down-regulated and less accessible in all three BM comparisons with CD138+ ASC, which is also evident in the accessibility of HRK between the blood and BM ASC subsets (Fig 4D and E).

### Late epigenetic regulation of pro-apoptotic loci in LLPCs

In contrast to the pro-apoptotic genes, pro-survival gene expression did not always accompany changes in chromatin accessibility when comparing blood and BM ASCs (Fig 4F). For example, *MCL1* followed this trend and increased its gene expression without concordant accessibility changes within the gene (Fig 4F and G). On the other hand, BCL2, CFLAR, and BIRC3 underwent concordant epigenetic and transcriptional changes (Fig 4F). Of these three, BCL2 was identified as having concordant changes in all blood and BM ASC comparisons, and this locus was more accessible and had higher expression in BM subsets (Fig 4F and G). However, among BM subsets, only BCL2 concordantly changed between Pop A versus D, suggesting that CD138- Pop A did not undergo the same chromatin accessibility changes as CD138+ BM ASC (Fig 4F and G). Indeed, the differences in BCL2 accessibility between ASC subsets highlighted by the analysis were also evident from the gene tracks (Fig 4G). Together, both pro-apoptotic and pro-survival epigenetic and transcriptional gene regulation is important in the initial blood to BM ASC changes, but the final maturation step for BM LLPCs is the chromatin remodeling of pro-apoptotic regions.

**Figure S5.**
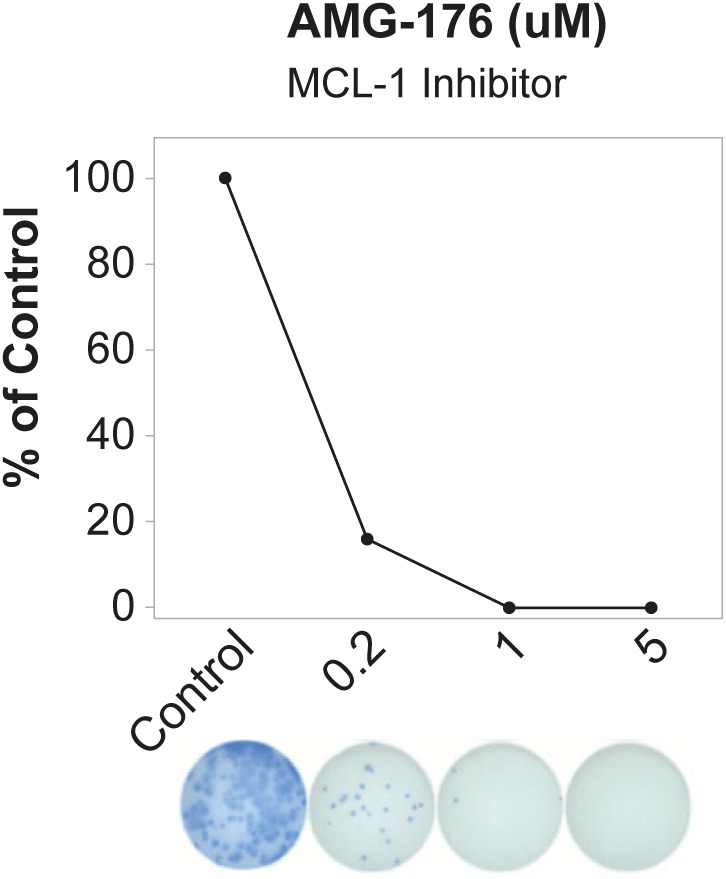
Titration of AMG-176 inhibitor, an MCL-1 inhibitor, using blood antibody secreting cells. Total IgG secretion of blood antibody secreting cell measured using an ELISPOT with or without exposure to different concentrations of AMG-176, an MCL1 inhibitor. Percent IgG graphed normalized to the number of spots in the control, which was untreated or treated with 0.1% DMSO.

To validate these changes, we selected MCL-1 as a target because we observed increased expression of *MCL1* in Pops B and D compared with blood ASCs and Pop A (Fig 4A and C). MCL-1 is a pro-survival protein that prevents apoptosis by interacting with BAK, thereby, minimizing mitochondrial outer membrane permeabilization (MOMP), which is an initial step in the intrinsic cell death pathway (Singh et al, 2019). We isolated blood and BM ASCs from adults after tetanus vaccination and steady state healthy adults (Table S1) and cultured the cells with and without AMG-176, a small molecule inhibitor of MCL-1, as previously described (Nguyen et al, 2018a, 2018b). We identified the IC_50_ of 0.2 μM by titrating in blood ASC (Fig S5). As predicted, blood ASCs and Pop A were sensitive to MCL1 inhibition, whereas Pop B and Pop D were resistant (Fig 4H). In sum, blood ASCs must undergo coordinated epigenetic and transcriptional changes to become apoptotic resistant and support their maturation into a LLPC.

### In vitro morphological, transcriptional, and epigenetic transformation of blood ASCs in the in vitro BM mimic

**Figure S6.**
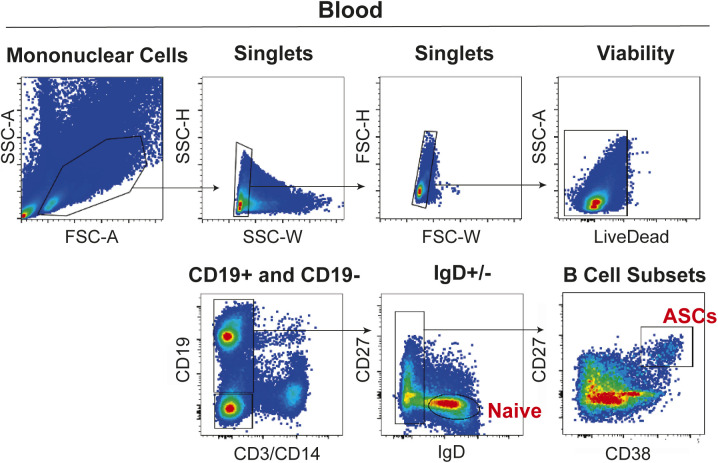
FACS gating strategy for obtaining blood antibody secreting cells for in vitro culture experiments. Fluorescence activating cell sorting strategy for enriching blood antibody secreting cell (CD3−C14−IgD−CD19+CD38+CD27++). Gating strategy for one representative sample from the peripheral blood of a 19-yr-old, F, Asian, non-Hispanic, healthy donor after vaccination is shown.

To test whether in vitro ASC maturation was similar to the transcriptional and morphological changes observed in the ex vivo blood and BM ASC, we sorted blood ASC (CD19+IgD−CD38+CD27+) from adults after tetanus vaccination and cultured them in the in vitro BM mimetic system (Fig S6; Nguyen et al, 2018b). Previous studies showed survival of nascent blood ASCs for 56 d in culture. The cells were harvested on 0, 1, 3, 7, or 14 d from the cultures for morphology, RNA-seq, and ATAC-seq (Table S1).

**Figure 5.**
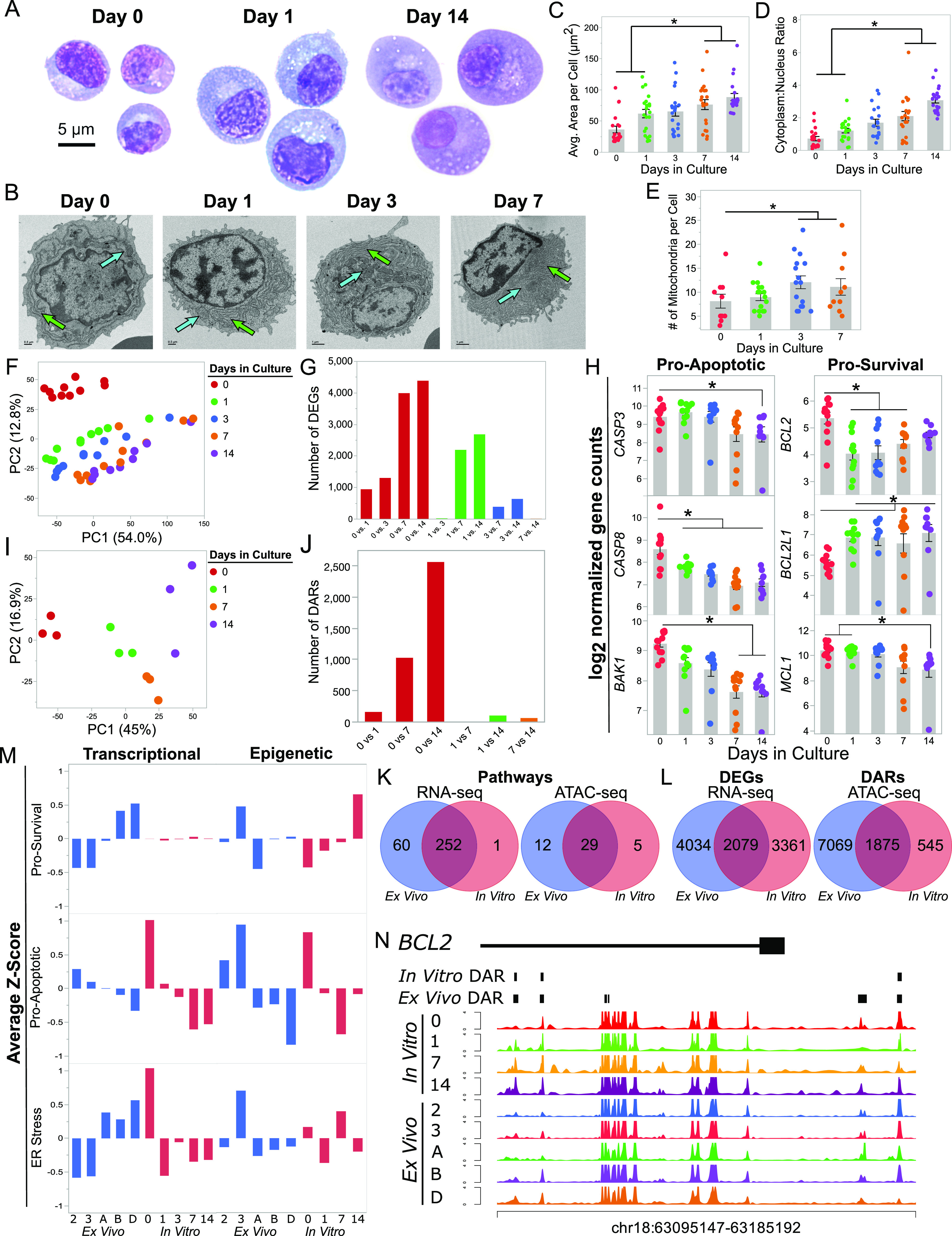
Human blood antibody secreting cells (ASCs) undergo morphological and transcriptional maturation in response to an in vitro BM mimic. **(A)** Representative Wrights-Giemsa images of human blood ASCs cultured for either 0, 1, or 14 d using cell-free bone marrow mesenchymal stromal cell (BMSC) derived secretome, APRIL and hypoxia. Scale bar is applicable to each population. **(B)** Transmission electron microscopy images of the indicated cell populations from two to three independent samples per population. Green and blue arrows indicate ER and mitochondria, respectively. Day 0 and 1 Scale Bar = 0.5 μm. Day 3 and 7 Scale Bar = 1 μm. **(C, D)** Comparison of the average area per cell (C) and cytoplasm to nucleus area ratio (D) after culturing cells in the BM mimic. Statistical significance was assessed using a linear mixed-effect model with Tukey–Kramer HSD post hoc analysis. **(E)** Quantification of the number of mitochondria per cell transmission electron microscopy images. Statistical significance was assessed using a generalized linear model using a background Poisson distribution with chi-square post hoc analysis. **(F)** Principal components analysis using differentially expressed genes (DEGs) identified between pairwise comparisons. **(G)** Bar graph indicating the number of DEGs per comparison. **(H)** Comparison of pro-survival (*BCL2*, *BCL2L1*, and *MCL1*) and pro-apoptotic (*CASP3*, *CASP8*, and *BAK1*) gene expression in ASC cultured in the BM mimetic. **(I)** Principal component analysis using differentially accessible chromatin regions (DARs) identified between pairwise comparisons. **(J)** Number of DARs by pairwise comparison of in vitro matured blood ASCs from days 0, 1, 7, and 14. **(K)** Venn diagrams of overlapping pathways of the DEGs and DARs between the ex vivo and in vitro systems identified by gene set enrichment analysis. **(L)** Venn diagram of overlapping DEGs and DARs between the ex vivo and in vitro systems. **(M)** Comparison of the average z-score of genes involved in ER stress, Pro-survival, and Pro-apoptotic processes as shown in Fig 4 between (blood Pop 2, 3, and BM Pop A, B, and D; Blue) and peripheral blood ASCs cultured for days 0–14 (Red). Left panel demonstrates the transcriptional changes and the right panel shows the epigenetic changes for (top) pro-survival, (middle) pro-apoptotic, and (bottom) ER stress related genes in the KEGG apoptosis pathway. **(N)** BCL-2 tracks from the ASC in vitro cultures on days 0–14 and ASC ex vivo (blood pop 2, 3, and BM pop A, B, and D). DARs of the in vitro and ex vivo data sets are shown at the top. Three overlapping DARs mapped to BCL-2. For all panels, asterisks indicate statistical significance, *P ≤ 0.05.

As expected, ASC subsets placed in the BM mimetic for 2 h on day 0 remain morphologically similar to blood ASCs Pop 2 and 3 ex vivo (Figs 1A and 5A). However, as early as 24 h later, the early minted ASCs began to increase their ER mass (Fig 5B). Although there was heterogeneity, the size generally increased consistently by day 14, and the cytoplasm to nucleus ratio increased significantly from baseline to 7 and 14 d in culture (Fig 5C and D). Interestingly, some cells increased in size and persisted to day 14, whereas the smaller cells with low cytoplasm to nuclear ratios disappeared (Fig 5D). The bigger cells persisted to day 14, whereas the smaller cells with low cytoplasm to nuclear ratios disappeared, suggesting heterogeneity of the ASC maturation process where cells that do not expand the ER may die. The later stages also had an increase in the number of mitochondria per cell compared to baseline (Fig 5E). These morphological and subcellular changes of the in vitro ASC maturation over 14 d reflected the changes between blood and BM ASCs ex vivo.

Transcriptomes of the ASCs matured in the in vitro BM mimetic cultures were similar to transcriptomes observed in the ex vivo analysis. The PCA analysis segregated samples based on their duration in the culture with the day 0 points clustering together followed by days 1, 3, 7, and then 14 (Fig 5F). Consistent with the PCA analysis, the largest pairwise comparisons occurred between ASC harvested from early points compared with later in culture (Fig 5G). As time progressed, the number of DEGs decreased substantially, indicating that the ASC transcriptomes stabilize after 7 d in culture (Fig 5G). Targeted analysis showed similar transcriptional changes with ex vivo samples in *CASP3*, *CASP8*, and *BAK1* expression which decreased with time (Fig 5H). *BCL2L1* expression continued to up-regulate the longer the cells were in the in vitro BM mimetic (Fig 4A). Although BM populations had increased *BCL2* and *MCL1* expression ex vivo, the in vitro ASCs did not up-regulate these genes by day 14 (Fig 5H).

To understand the epigenetic maturation programs of cultured ASCs in the BM microniche, we examined the ATAC-seq profiles of early minted ASCs from day 0 to 14 in three subjects. PCA analysis showed the primary driver of variance was how long the cells were in culture with the greatest pairwise comparisons of DARs between day 0–7 and 0–14 (Fig 5I and J). Transcriptionally and epigenetically, the in vitro system mirrors the changes in maturation observed within our ex vivo populations. There are 252 (or 80.5%) overlapping pathways enriched between the DEGs and 29 (or 63%) overlapping pathways enriched between the DARs of the two systems (Fig 5K). Whereas there were fewer pathways that overlapped with DARs, there were a similar number of DEGs and DARs that overlapped in the RNA-seq and ATAC-seq analysis of the maturation models (2,079 shared DEGs, 1,875 shared DARs; Fig 5L). Importantly, the overlapping transcriptional and epigenetic programs between the ex vivo and in vitro ASC datasets demonstrates that many changes occur as early as 7–14 d. Overlapping transcriptional and epigenetic pathways, as well as DEGs and DARs, of the ASCs matured in vitro demonstrate similar LLPC maturation programs as the ex vivo blood to BM LLPCs. Thus, our BM mimetic system not only maintains ASC survival but also transforms early minted ASC to become LLPC morphologically, transcriptionally, and epigenetically. These results are akin to the differences between ex vivo blood and BM LLPCs.

Using the average Z-score of the DEGs in the pro-survival, pro-apoptotic, and the ER stress pathways to assess trajectory, we demonstrate that the ASCs in the in vitro cultures had similar trends with the changes of ASCs ex vivo (Fig 5M). As expected, the RNA-seq showed decreased pro-apoptotic trajectories and increased pro-survival gene BCL2L1 of ASCs ex vivo and in vitro. For the ATAC-seq, gradual increases in the pro-survival trajectories occurred over the 14-d cultures and the inaccessibility of pro-apoptotic regions required at least 7 d (Fig 5M). Of the 11 BCL-2 DARs from the ex vivo blood and BM populations (Fig 4G), three sites were differentially accessible chromatin regions (DARs) in our in vitro dataset (Fig 5N) suggesting the unique and novel transcriptional and epigenetic regulation and importance of BCL-2 in the maturation of human LLPCs. Furthermore, the BM mimetic system is a novel tool that facilitates not only the morphological changes involved in maturation, but also the concordant transcriptional and epigenetic changes associated with maturation. In conclusion, these results demonstrate that the human LLPC maturation programs include not only the transcriptional changes but also require further epigenetic imprinting to become a LLPC. In all, the BM microniche drives the morphological, transcriptional, and epigenetic changes necessary for LLPC maturation from an early-minted ASC and suggests that there is heterogeneity in blood ASC ability to respond and establish in this environment.

### CD138+ BM ASC persist for at least 1 yr

**Figure 6.**
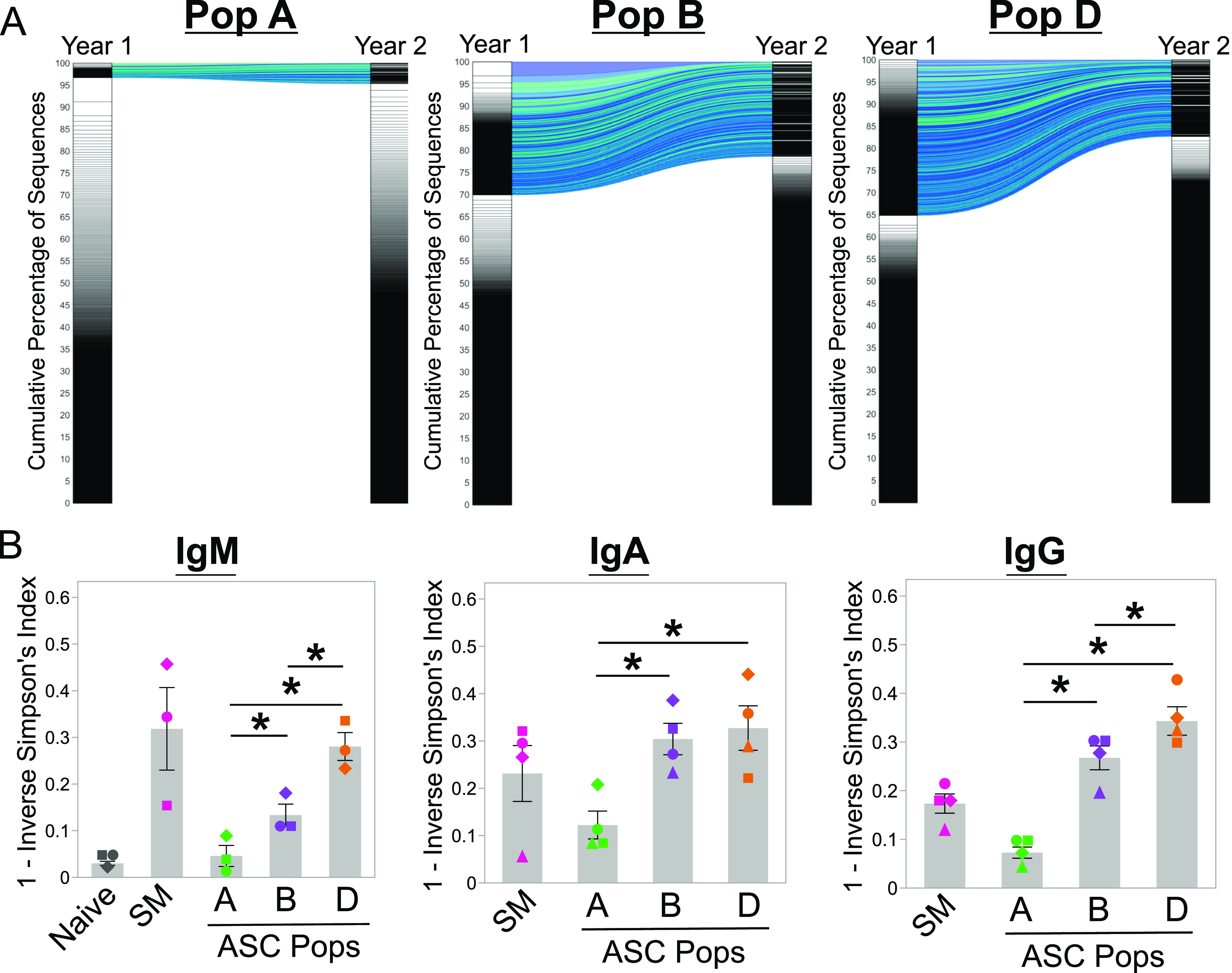
Assessment of the persistence of human blood and BM antibody secreting cell populations using next-generation sequencing. **(A)** Alluvial plots showing the connectivity of clones after sequentially sampling BM populations A, B, and D from a healthy donor at Year 1 and Year 2. The diversity of the repertoire is shown by plotting lineage (clone) size versus the cumulative percentage of sequences determined from size-ranked clones from Year 1 to Year 2 is shown in Pop A, Pop B, and Pop D. Largest clones are found at the top of the plot and account for a greater area within the subdivided plots. More diverse repertoires, such as BM population A here, only contain small clones in a more even representation. **(B)** The diversity of repertoire by isotype shown across five BM populations: naïve, switched memory, Pop A, Pop B, and Pop D. Diversity is expressed as 1—the Inverse Simpson’s Index. Statistical significance was assessed using a linear mixed effect model with population as the fixed effect and patient as a random effect. Asterisks indicate statistical significance, *P ≤ 0.05. All figures used VDJ sequencing data obtained from patient 503, which is representative of the four patients in the analysis.

To evaluate persistence of ASC clones in the BM subsets, we compared bulk VDJ sequencing as previously described (Halliley et al, 2015) from BM Pops A, B, and D of four healthy adults and then repeated BM aspirates 1 or 2 yr later. More than 30% of ASC clones identified within Pops B and D persisted over time with slightly more in Pop D. Less than 5% of the lineages in Pop A persisted from year to year (Fig 6A). Using the 1-inverse Simpson index to measure similarities in two populations from year to year, we divided them by isotype. As expected, naïve B cells which contained IgM sequences showed little connectivity over the course of 1 yr indicating polyclonality and high turnover, whereas circulating memory cells were more persistent (Fig 6B). Within the BM, IgM+ ASCs were most persistent within Pop D LLPC, whereas Pop A showed little longitudinal connectivity. Class-switched (IgG+ and IgA+) ASCs persisted in both the Pop B and D from year to year, whereas Pop A once again displayed little connectivity over the 1-yr period. Combined, our results indicate that both Pop B and D have a high shared cumulative percentage of sequences indicating a persistence of clones for at least 1–2 yr. If we had followed BM clones over 5–10 yr, we may have noted differences between pop B and D. In all, this result demonstrates clones in CD138+ ASC in the BM persist over 1 yr.

## Discussion

Although it is well established that human LLPC reside in the BM, the debate continues whether LLPC merely migrate to their survival niches and take up residence or undergo further transformation once in the BM locale. In this study, we examined the morphology, transcriptional and epigenetic programs between the blood and BM ASC subsets ex vivo and showed that early-minted ASCs change morphologically, transcriptionally, and epigenetically as they mature into an LLPC. The ex vivo ASC comparisons show that this process involves acquiring a phenotype that becomes refractory to apoptosis in a developmentally programmed fashion transcriptionally and epigenetically. Additional validation of the maturation process with our novel in vitro human BM mimetic cultures demonstrate similar morphologic, transcriptional, and epigenetic transformation of the same early minted ASC into LLPC phenotype.

The transcriptional changes as B cells differentiate into ASCs have been well-described (Shapiro-Shelef et al, 2003; Klein et al, 2006; Sciammas et al, 2006; Minnich et al, 2016; Tellier et al, 2016). In contrast, this study illustrates how the BM microniche further matures nascent ASCs into LLPCs. This process requires both the early down-regulation of pro-apoptotic genes (*BAK1*, *BAX*, *CASP3*, and *CASP8*) and up-regulation of pro-survival genes (*MCL1*, *BCL2*, and *BCLXL*), although timing may be different for some of the pro-survival genes. *BCL-xL* (*BCL2L1*) up-regulation occurs early, whereas *BCL2* and *MCL1* up-regulation may happen later in maturation. Although the apoptotic pathway is modified transcriptionally, the LLPC maturation process also involves chromatin accessibility changes that arise later. These results show that LLPC acquire chromatin modifications that may contribute to apoptotic resistance, and our results demonstrate that the BM microniche factors trigger some of these changes.

Despite the modest number of DEG between the CD138+ BM subsets, the high numbers of DARs (3,452) between pop B and D demonstrate that epigenetic modifications are the hallmark of LLPC maturation. To our surprise, epigenetic regulation of multiple pro-apoptotic loci was found to be invaluable for this maturation process, whereas only the *BCL2* locus among the anti-apoptotic loci was most significant. We and others had previously reported the importance of *BCL2* and *MCL1* in the transcriptional regulation of LLPC (Halliley et al, 2015; Mei et al, 2015); but, the importance of *BCL2* and pro-apoptotic chromatin regulation in the human LLPC are new mechanisms for the last step of the LLPC maturation process. Further studies will be needed to define the temporal sequence of the maturation steps.

LLPCs have higher expression of *MCL1* and become more resistant to the MCL1 inhibitor, AMG-176. Our results agree with mouse studies that showed that *MCL1* is essential for BM PC survival (Peperzak et al, 2013). Activation of BCMA by APRIL increases expression of the anti-apoptotic molecule *MCL1*, indicating a potential mechanistic role for the APRIL/BCMA axis to promote long-term survival. Again, we had also reported that human blood and BM ASC have increased surface expression of BCMA (Halliley et al, 2015; Garimalla et al, 2019) with further validation that APRIL is a critical PC survival factor that is not provided by primary BM stromal cells (unpublished data). Clearly APRIL:BCMA interaction is important and responsible for *MCL1* up-regulation ex vivo because *MCL1* was highly expressed within 2 h of APRIL exposure in the culture. Its role together with BCL-2 and BCL-xL in transcriptional or epigenetic modifications in our in vitro models may require longer cultures and single cell resolution due to heterogeneity.

Within the BM, the CD138+ ASC (Pop B and D (LLPC)) were transcriptionally similar with only 85 DEGs. Unsurprisingly, both of the CD138+ BM ASC compartments show persistence of the same VDJ clones after 1 yr even though it was statistically higher in pop D for IgM and IgG isotypes. Because LLPCs (Pop D) contained viral specificities from exposures 40 yr ago (Halliley et al, 2015), we expected lineages to persist in this compartment longer than any other. There may be several reasons why lineages pop B and D after 1–2 yr were quite similar. The first may have been an issue with limited sampling of the clones with the maximal volumes taken for research BM aspirates. Second, clones in Pop D may persist longer than in Pop B if we had waited long enough; thus, follow-up in 5 yr may have demonstrated greater differences. Last, clones in Pop B may eventually mature into Pop D on an ongoing basis. In all, persistence of clonal lineages in CD138+ BM ASC compartments from year to year illustrates the vital importance of the BM microniche for survival.

We found little transcriptional and epigenetic differences between the two largest ASC subsets in circulation Pop 2 and 3 after tetanus vaccination similar to our previous transcriptional results (Garimalla et al, 2019). Others reported similar findings of influenza-specific ASCs after influenza vaccination on a single cell level yet they noted differences between non-influenza–specific IgA ASC (Neu et al, 2019). In healthy individuals at steady state, a predominance of IgA ASCs circulate, whereas during vaccination, IgG ASCs dominate (Mei et al, 2010), and our studies with early minted blood ASC subsets showed that each subset (using CD19, CD38, CD27, and CD138) had equal potential for survival in the BM mimetic cultures (Garimalla et al, 2019). Thus, steady-state IgA ASCs may not persist as well as vaccine-specific IgG ASCs. Despite these conclusions, not all IgG ASCs survive in these cultures equally suggesting the intrinsic cues permit a fraction to mature. Nonetheless, we show that the BM microniche provides important extrinsic factors, but it is also likely that both intrinsic fates of nascent ASCs together with the BM extrinsic factors are important for becoming an LLPC.

The blood populations, Pop 2 and 3, had a relatively small number of DARs when compared with the BM Pop B. Prima facie this suggests that these blood populations need fewer chromatin alterations as they migrate from the blood into the BM. A caveat to this interpretation is that assessment of statistical significance is a function of the heterogeneity in the populations, and the low apparent divergence is likely in part a consequence of considerable cell-to-cell variability in Pop B. Pop B may contain few blood ASCs as new immigrants (Pop 2 and 3) as well as lineages that have already persisted for 1 yr or more. Thus, more granular single cell resolution will be required to illustrate how chromatin remodeling shapes the maturation of blood ASCs which are poised to migrate from the blood to the BM to become LLPCs.

Although we emphasized apoptosis, there were many additional pathways identified in the LLPC maturation process. Some included signaling pathways such as mTOR, NF-κ B, TNF, and FoxO were prominent in transcriptional regulation. Consistent with our previous finding, down-regulation of mTOR pathways in LLPCs was associated with increased resistance to traditional mTOR inhibitors, rapamycin and sirolimus in the LLPC compared with early blood ASCs (Nguyen et al, 2018a). Many transcriptional pathways were concordant with the epigenetic modifications such as lysosome, phagosome, and autophagy highlighting the influence of mTOR down-regulation with autophagy and lysosomal degradation to recycle misfolded proteins in LLPC as mouse models had previously revealed (Pengo et al, 2013). Other concordant pathways were identified such as *VEGF*, *WNT*, and inositol phosphate, all of which warrant additional studies to appreciate their roles in survival, metabolism, and regulation of Ig secretion.

We chose to study ASC after tetanus toxoid vaccination which is known to maintain serum antibody half-life of 10 yr and is consistent with a long-lived vaccine. Because these vaccines are universally administered in childhood, we exclusively studied ASCs from memory B cells in healthy adults (Halliley et al, 2010; Lee et al, 2011). ASC kinetics from naïve B cells after primary immunization occurs on days 12–14 (Blanchard-Rohner et al, 2009) and was not included in this study. LLPCs have traditionally been thought to come from secondary immunizations and memory B-cell origins. Regulation of apoptosis in well-defined memory subsets provide valuable insights into intrinsic cues of ASCs derived from memory B-cell origins (Lau et al, 2017). For example, memory B cells have increased expression of TNFR superfamily members (TNFSF ligands and TNFRSF receptors) and SLAM family receptors (Good et al, 2009). In addition, switched memory B cells and ASC have an increased expression of *MCL1* (Vikstrom et al, 2010; Peperzak et al, 2013). The observed increased expression of *BCL2*, *BCL2A1*, and *MCL1* in memory B cells likely contributes to their improved survival in vitro compared to naïve B cells. Whether memory B cells have chromatin accessibility promoting pro-survival pathways will provide insights of the roadmap to becoming an LLPC.

In summary, peripheral blood ASC subsets are morphologically, transcriptionally, and epigenetically distinct from BM populations, and the BM microniche provide important factors and conditions that promote maturation to an LLPC phenotype. The choreographed down-regulation of pro-apoptotic genes and up-regulation of pro-survival genes together with the chromatin modifications of the apoptosis pathway is essential to become a human LLPC.

## Materials and Methods

### Subjects

Blood was obtained from 36 healthy adult subjects (age 63–18, mean 27.5 ± 12.98 [SD]) that had been recently vaccinated with Tdap, Shingrix, PSV23, HepA, or HepB vaccines. All vaccines were administered as part of standard medical care. BM aspirates were obtained from 30 immunologically healthy individuals (age 63–19, mean 40.4 ± 14.8 [SD]). A supplemental table containing the information for each patient used in the study is provided in Table S1. Patients were recruited between 2008 and 2021 at the University of Rochester or Emory University. All experiments were performed from fresh blood and BM samples; no frozen samples were used in this study. All studies were reviewed and approved by the Institutional Review Board at the University of Rochester and Emory University. IRB Approval numbers were 11935 at the University of Rochester and 66294, 58507, and 57983 for Emory University.

### Blood and BM ASC isolation

PBMCs and BM mononuclear cells were isolated from fresh blood and BM aspirate samples, respectively, by density gradient centrifugation using Lymphocyte Separation Medium (Cellgro/Corning). After isolation, the mononuclear cell fractions were further enriched by negative selection using a MACS column that removed CD3+/CD14+ cells, a commercial human pan-B cell enrichment kit that removes cells expressing CD2, CD3, CD14, CD16, CD36, CD42b, CD56, CD66b, CD123, and glycophorin A, or a custom stem cell kit that removes CD66b+/GPA+/CD3+/CD14+ cells (StemCell Technologies). Enriched fractions were then stained using the fluorescently conjugated antibodies IgD–FITC (Cat. no. BD555778; BD Biosciences), CD3-BV711 (Cat. no. 317328; BioLegend), CD14-BV711 (Cat. no. 301838; BioLegend), CD19-PE-Cy7 (Cat. no. 301838; BD Biosciences), CD38-V450 (Cat. no. BDB561378; BD Biosciences), CD138-APC (Cat. no. 130-117-395; Miltenyi Biotech), CD27-APC-e780 (Cat. no. 5016160; eBiosciences), and LiveDead (L34966; Invitrogen) and populations of ASC were purified by fluorescent activated cell sorting for further experiments. Cell sorting experiments were performed on a FACS Aria II (BD Biosciences) using a standardized sorting procedure that used rainbow calibration particles to ensure consistency of sorts over time. The gating strategies used to isolate ASC populations are in Figs S1 and S6.

### Wrights-Giemsa

500–20,000 cells were adhered to positively charged microscope slides by centrifugation at 41*g* for 5 min at RT using a cytospin. Slides were then dried and fixed with methanol followed by staining with a 1–4% Wrights-Giemsa solution for 20 min at RT. After staining, slides were rinsed, dried, and imaged at 1,000× using a Zeiss microscope. Cellular features were analyzed using ImageJ (ImageJ 1.52q, https://imagej.nih.gov/ij/). Cell area was calculated by using the freehand area selection tool, performing five independent measurements around the cellular membrane, and averaging those values together. The nuclear area was calculated by using the area selection function in ImageJ. Five independent measurements were taken of the nucleus area and averaged together. The area of the cytoplasm was calculated by subtracting the average area of the nucleus from the cell area. The nucleus to cytoplasm ratio was calculated by dividing the average area of the nucleus by the area of the cytoplasm of each cell.

### TEM

FACS-purified ASCs were pelleted by centrifugation at 500*g* for 5 min at 20°C, and the supernatant was removed by aspiration. The pellet was then resuspended with ∼1 × 10^6^ erythrocytes in phosphate-buffered saline. The addition of erythrocytes was necessary to visualize the pellet during TEM processing. The combined pellet was then fixed with 2.5 M glutaraldehyde at 4°C overnight. The pellet was then placed into 0.1% osmium tetroxide in 0.1 M phosphate buffer (pH 7.4) for 1 h, followed by dehydration in sequential incubations in 25%, 50%, 75%, 95%, and 100% ethanol solutions. The pellet was infiltrated, embedded, and polymerized in Eponate 12 resin (Ted Pella Inc.). Sections of ∼70 nm thickness were cut using a Leica Ultracut S ultramicrotome and stained with 5% uranyl acetate and 2% lead citrate before imaging. The TEM grids were then imaged using a JEOL JEM-1400 TEM (JEOL Ltd.) with Gatan US1000 2k × 2k CCD camera (Gatan).

### RNA-seq sample preparation and analysis

Purified ASC populations for ex vivo analyses were sorted directly into RLT buffer containing 1% 2-Mercaptoethanol. ASC harvested after incubation in the in vitro BM mimic were pelleted by centrifugation at 500*g* at 20°C then resuspended in RLT buffer containing 1% 2-Mercaptoethanol. Total RNA was isolated from all samples using the Quick-RNA Microprep kit (Zymo Research), and all resulting RNA was used as input for the SMART-seq v4 cDNA synthesis kit (Takara) with 12 cycles of PCR amplification. cDNA was quantified by Qubit, and 200 pg of material was used to generate final sequencing libraries with the NexteraXT kit and NexteraXT Indexing primers (Illumina, Inc) using 12 cycles of PCR amplification. After library preparation, all libraries were quality-checked on a bioanalyzer, quantitated by Qubit fluorometer, and pooled at equimolar ratios before sequencing on a NextSeq500 using 75 bp paired-end chemistry. Sequencing was performed using a NextSeq500 instrument at the University of Alabama Birmingham Helfin Genomics Core or a NovaSeq6000 at Novogene. All samples harvested for ex vivo analysis or in vitro experiments using the BM mimic were prepped and sequenced together to minimize batch effects.

### RNA sequencing data analysis

For ex vivo analysis, raw sequencing files were processed using Partek Flow Genomics Suite (Partek Inc.). The average number of reads across all sample populations was between five and six million, and the average PHRED quality score for each base pair was between 33 and 35 after trimming. Trimmed reads were aligned to the hg38 version of the human genome followed by quantification using the union model in HTSeq with default settings. After alignment, low expression genes with <150 counts for a given gene across all samples were removed followed by library-size normalization using the rlog function in DESeq2 with default parameters. After normalization, data were visualized using principal component analysis to detect outliers. If outliers were observed, these samples were removed, and the data were re-normalized before further analysis. For ex vivo analyses, there were 11 Pop 2, 11 Pop 3, 10 Pop A, 11 Pop B, and 10 Pop D samples that were high quality and used in our analyses.

For in vitro experiments with the BM mimic, raw sequencing files were mapped to the hg38 genome using STAR (PMID: 23104886) with the default settings and the Gencode v27 reference transcriptome. Reads mapping to exons were summarized for all unique ENTREZ genes using the GenomicRanges (PMID: 23950696) package in R v3.3.6. Genes with <1,000 counts for a given gene across all samples were removed followed by library-size normalization using the median ratio function in Partek Flow Genomics Suite (Partek, Inc.). Outliers were detected after normalization using a PCA plot and were removed, and data were re-normalized prior to analysis. There were 12 Day 0, 11 Day 1, 10 Day 3, 11 Day 7, and 9 Day 14 samples from the in vitro dataset that were of high quality and used in our analyses.

The normalized data for both data sets were analyzed using Partek Genomic Suite. Differential gene expression analysis was performed with a linear-mixed effect model with the population as a fixed effect and patient as a random effect. A Tukey–Kramer post hoc analysis was used for pairwise comparisons. *P*-values for all comparisons were false discovery rate (FDR) corrected by the method of Benjamini–Hochberg. A gene was considered differentially expressed for ex vivo analyses if the pairwise comparisons had an FDR-corrected *P*-value of less than 0.05. A gene was considered differentially expressed for in vitro analyses if the pairwise comparisons had a fold change > 2 and an FDR-corrected *P*-value of less than 0.05 between at least one comparison. Pathway analyses using all genes were performed using Partek’s genomic suites algorithm with the KEGG database. Pathways with an FDR-corrected *P*-value of less than 0.05 were considered statistically significant.

### ATAC sequencing

ATAC-seq was performed as previously described (PMID: 27249108). Briefly, FACS isolated cells were resuspended in 25 ml tagmentation reaction buffer (12.5 ml Tagment DNA Buffer; Illumina, Inc, 2.5 ml Tn5, 0.02% Digitonin, 0.1% Tween-20) and transposed at 37°C for 1 h. Transposed nuclei were lysed by addition of 2× in lysis buffer (300 mM NaCl, 100 mM EDTA, 0.6% SDS, and 1.6 mg Proteinase-K), and incubated for 30 min at 40°C. Size selection using SPRI-beads isolated low molecular weight DNA which was then PCR amplified using 2 × HiFi HotStart Ready mix (Roche Diagnostics) and Nextera Indexing Primers (Illumina, Inc). A second size-selection was performed post-PCR to enrich for low molecular weight DNA. Samples were quality checked for ATAC-seq specific patterning on a bioanalyzer and were pooled at an equimolar ratio and sequenced on a NextSeq500 using 75 bp paired-end chemistry at the University of Alabama, Birmingham Helfin Genomics Core.

### ATAC sequencing data analysis

Raw sequencing reads were mapped to the hg38 version of the human genome using Bowtie2 v2.2.4 (Langmead & Salzberg, 2012) and duplicate reads flagged using PICARD (http://broadinstitute.github.io/picard/) filtered based on the uniquely mappable and non-redundant reads. After sequencing, 6 Pop 2, 7 Pop 3, 7 Pop A, 9 Pop B, and 6 Pop D samples (ex vivo), and 3 Day 0, 3 Day 1, 3 Day 7, and 3 Day 14 samples (in vitro) passed quality control and were used for analysis. Enriched peaks were determined using MACS2 v2.1.0.2014061 (Zhang et al, 2008) and the reads for each sample overlapping all possible peaks was calculated using the GenomicRanges v1.34.0 (Lawrence et al, 2013) package in R v3.5.2. Differential accessible regions/peaks (DAR) were determined using edgeR v3.24.3 (Robinson et al, 2010) and peaks that displayed an absolute log_2_ fold change (log_2_FC) > 1 and a Benjamini–Hochberg FDR corrected *P*-value < 0.05 were considered significantly different.

### In vitro culture using BM mimic and inhibitor assays

In vitro cultures of human blood and BM plasma cells were performed as described (Nguyen et al, 2018a, 2018b). Blood ASCs and BM plasma cell populations were cultured in cell-free mesenchymal stromal cell secretome media in 96-well flat bottom cell culture plates (Corning/Sigma-Aldrich) at 37°C in a humid, 5% CO_2_, 95% air (20% O_2_) incubator, or in hypoxic culture conditions (2.5% O_2_) at 37°C in a cell culture incubator programmed for the desired O_2_ tension. Titration curves were established to find the half maximal inhibitor concentration (IC_50_) for AMG-176, and subsequent experiments were performed using a concentration of 0.2 μM. Survival and antibody secretion of blood ASCs and BM populations were assessed using ELISpot assays as previously described (Nguyen et al, 2018a, 2018b; Garimalla et al, 2019). Survival and function were expressed as the percentage IgG-secreting cells normalized to the control.

### Repertoire analysis

VH next generation was conducted on paired human BM samples. The first BM sample was drawn from each donor, and a second longitudinal sample was drawn after 1 yr. Total cellular RNA was isolated from: Pop A, B, D from four matched BM samples using the RNeasy Mini Kit (QIAGEN, Inc) by following the manufacturer’s protocol. Approximately 400 pg of RNA was subjected to reverse transcription using the iScript RT kit (BioRad, Inc.). Resulting cDNA products were included with 50 nM VH1-VH6 specific primers and 250 nM Ca, Cm, and Cg specific primers in a 20 μl PCR reaction using High Fidelity Platinum PCR Supermix (Life Technologies) and amplified by 40 cycles. Nextera indices were added and products were sequenced on an Illumina MiSeq with a depth of ∼300,000 sequences per sample. All sequences were aligned with IMGT.org/HighVquest (Alamyar et al, 2012). Sequences were then analyzed for V region mutations and clonality. All clonal assignments were based on matching V and J regions, matching CDR3 length, and 70% CDR3 homology. All sequences are plotted using MATLAB or Circos visualization tools (Krzywinski et al, 2009).

## Data Availability

The RNA- and ATAC-sequencing data from this publication have been deposited to the NCBI GEO database under accession GSE180090. The repertoire sequencing data from this publication have been deposited to the Sequence Read Archive under accession ID PRJNA716430. All other raw data can be requested from the corresponding author.

## Supplementary Material

Reviewer comments
